# The Influence of Aging on the Regenerative Potential of Human Adipose Derived Mesenchymal Stem Cells

**DOI:** 10.1155/2016/2152435

**Published:** 2016-01-28

**Authors:** Monika Marędziak, Krzysztof Marycz, Krzysztof A. Tomaszewski, Katarzyna Kornicka, Brandon Michael Henry

**Affiliations:** ^1^Department of Animal Physiology and Biostructure, Faculty of Veterinary Medicine, Wroclaw University of Environmental and Life Sciences, 50-375 Wroclaw, Poland; ^2^Electron Microscopy Laboratory, Wroclaw University of Environmental and Life Sciences, 50-631 Wroclaw, Poland; ^3^Wroclaw Research Centre EIT+, 54-066 Wroclaw, Poland; ^4^Department of Anatomy, Jagiellonian University Medical College, 31-034 Krakow, Poland

## Abstract

Tissue regeneration using human adipose derived mesenchymal stem cells (hASCs) has significant potential as a novel treatment for many degenerative bone and joint diseases. Previous studies have established that age negatively affects the proliferation status and the osteogenic and chondrogenic differentiation potential of mesenchymal stem cells. The aim of this study was to assess the age-related maintenance of physiological function and differentiation potential of hASCs in vitro. hASCs were isolated from patients of four different age groups: (1) >20 years (*n* = 7), (2) >50 years (*n* = 7), (3) >60 years (*n* = 7), and (4) >70 years (*n* = 7). The hASCs were characterized according to the number of fibroblasts colony forming unit (CFU-F), proliferation rate, population doubling time (PDT), and quantified parameters of adipogenic, chondrogenic, and osteogenic differentiation. Compared to younger cells, aged hASCs had decreased proliferation rates, decreased chondrogenic and osteogenic potential, and increased senescent features. A shift in favor of adipogenic differentiation with increased age was also observed. As many bone and joint diseases increase in prevalence with age, it is important to consider the negative influence of age on hASCs viability, proliferation status, and multilineage differentiation potential when considering the potential therapeutic applications of hASCs.

## 1. Introduction

Mesenchymal stem/stromal cells (MSCs) hold great promise as a novel therapeutic option for use in tissue regeneration. Because of their multilineage differentiation capacity, MSCs are considered as a potential therapeutic tool for treating a wide range of pathologies, especially bone and cartilage disorders such as osteoarthritis, osteoporosis, and osteonecrosis [1–3]. Numerous preclinical and clinical studies, using the ability of MSCs to repair bone and cartilage, have shown promising results in musculoskeletal regeneration of chondral and osteochondral lesions [4–7]. This lends hope for MSCs to be potentially used in daily clinical practice.

Beside proliferative and multilineage differentiation potential, MSCs have an immunomodulatory effect that is dependent on cell-cell contact or mediated through the secretion of immunosuppressive molecules [8, 9]. The ability of MSCs to secrete membrane derived vesicles (MVs), rich in a wide range of growth factors, antiapoptotic factors, and anti-inflammatory molecules, is currently considered as a novel molecular mechanism with significant therapeutic potential [10, 11]. Multipotent cells have been isolated from many sources including adipose tissue, bone marrow, and the umbilical cord [12, 13]. Traditionally, human MSCs are isolated from an aspirate of bone marrow harvested from the iliac crest or the acetabulum. However, the most common and effective way of acquiring the cells is from adipose tissue [14, 15]. Adipose derived mesenchymal stem cells (ASCs) are easily obtained from patients during surgery or through minimally invasive procedures. Results from a number of animal studies have confirmed that ASCs can be successfully used to treat musculoskeletal defects in vivo [16–18].

In humans, the most common disease of the joints is osteoarthritis (OA) [19]. The prevalence of OA increases with age and patients with obesity are at increased risk [20]. Moreover, articular cartilage injuries, which originate from fractures and dislocations, lead to the development of posttraumatic osteoarthritis (PTOA) [21]. Due to articular pain, PTOA causes decreased joint function and disability [21]. Clinical and epidemiologic experience indicates that prevalence of PTOA after joint surface fracture is strongly dependent on a patient's age. Adults in their 20s and 30s are significantly less likely to develop PTOA in injured joints as compared to adults over the age of 50. Although cartilage displays a very limited capacity for self-repair, it is believed that MSCs might be the key to complete cartilage regeneration [22, 23].

Studies conducted over the past few years have suggested that aging impacts various MSC properties [24, 25]. This effect is most clearly illustrated by changes in two specific parameters: osteogenic differentiation and proliferation [12, 26–28]. However, there is evidence to suggest that the amount of MSCs, their proliferation rate, and population-doubling potential diminish with a patient's age. Moreover, an age dependent correlation between the proliferative potential of MSCs and cell apoptosis has been described [29]. Similar effects have been reported for periosteal progenitor cells and hematopoietic stem cells [30, 31]. These findings suggest that the regenerative potential of MSCs is downregulated with age and points to a possible limitation in their potential therapeutic use.

In order to draw a conclusion on the age-related maintenance of physiological function and differentiation potential of human ASCs in vitro, this study assessed several parameters: the number of fibroblasts colony forming unit (CFU-F), proliferation rate, population doubling time (PDT), and quantified parameters per lineage for osteogenic, adipogenic, and chondrogenic differentiation. This allowed us to further address a possible interdependence between viability and osteogenesis, adipogenesis, and chondrogenesis of MSCs.

## 2. Materials and Methods

To study the influence of age on the viability, morphology, and in vitro differentiation potential of human ASCs (hASCs) cells, the cells from individuals >20, >50, >60, and >70 years old were investigated. Mesenchymal stem cells were characterized for proliferation rate and CFU-F, along with measurements of population doubling time (PD), superoxide dismutase (SOD) activity, cellular senescence, apoptosis, and differentiation potential.

### 2.1. Experimental Groups

In subsequent experiments, cells were recruited and divided into four groups based on the donor's age, both male and female healthy subjects (the number of males and females was equal): (1) >20 years (mean age 24 ± 1.4 years; *n* = 8), (2) >50 years (mean age 57.5 ± 0.7 years; *n* = 8), (3) >60 years (mean age 67 ± 1.4 years; *n* = 8), and (4) >70 years (mean age 75 ± 2.8 years; *n* = 8).

### 2.2. Cell Collection and Isolation

All cell handling procedures described herein were performed in accordance with the ethical standards laid down in the 1964 Declaration of Helsinki and its later amendments and were approved by the Local Bioethics Committee of Wroclaw Medical University (registry number KB-177/2014). Written, informed consent for using tissue samples for research purposes was obtained from each and every patient prior to surgery.

Human mesenchymal stem/stromal cells were isolated from subcutaneous adipose tissue. Patients' fat biopsies (patient age ranged from 22 to 77 years) were obtained during total hip/knee arthroplasty or other open procedures connected with fracture reduction and fixation. Samples were placed in Hanks' balanced salt solution (HBSS) (Sigma-Aldrich, Germany) and transferred to the laboratory. According to a standard protocol [32], tissue samples were washed two times in HBSS with 1% antibiotic solution (Penicillin/Streptomycin/Amphotericin b, Sigma-Aldrich, Germany) and separated using surgical scissors. The minced tissue was digested in collagenase type I solution (1 mg/mL, Sigma-Aldrich, Germany) for 40 minutes at 37°C. After centrifugation (1200 ×g, 10 min) the supernatant was discarded and the pellet containing the cells was resuspended in culture medium and placed in a cell culture flask.

### 2.3. Cell Culture

Cells were maintained at constant conditions in an incubator (37°C, 5% CO_2_ and 95% O_2_) throughout the experiment. The primary culture was plated in a T-25 culture flask and cultured on Dulbecco's Modified Eagle's Medium (DMEM, Sigma-Aldrich, Germany) with nutrient F-12 Ham, 10% of Fetal Bovine Serum (FBS, Sigma-Aldrich, Germany), and 1% antibiotic solution and then transferred to a culture flask. The primary hASC culture was designated “passage 0.” The culture medium was changed every three days until the cells reached approximately 80% confluence. Adherent cells were detached from the flask using TrypLE*™* Express (Life Technologies, Poland). Cells were passed three times before starting the experiments. For viability, 7-day test cells were cultured in DMEM/Ham's F12 medium, supplemented with 10% FBS and with 1% antibiotic solution.

For chondrogenic, osteogenic, and adipogenic differentiation experiments, hASCs were cultured in STEMPRO® Adipogenic, Osteogenic and Chondrogenic Differentiation Kits (STEMPRO, Life Technologies, Poland).

The test cells were maintained in 24-well plates and inoculated at concentration of 30 × 10^3^ cells per well. The media was changed every two days. Chondrogenic and osteogenic stimulation was conducted for 21 days, whereas cells were cultured in adipogenic medium for 14 days.

### 2.4. hASC Characterization

The purity of MSCs was verified using fluorescent-activated cell sorting (FACS). The absence of the hematopoietic marker CD34 and the lymphocyte common antigen CD45, the presence of mesenchymal markers (CD90, CD73b, CD44, and CD29), and the cell's ability to differentiate into chondroblasts, osteoblasts, and adipoblasts confirmed that the obtained cells were in fact MSCs.

### 2.5. hASCs Clonogenic and Proliferation Potential

The clonogenic potential of hASCs from each group was tested by colony forming unit-fibroblastic (CFU-F) assay. The cells were seeded at density 1*∗*10^3^ cells/well and the medium was changed every 3 days. Cell clusters of more than 50 cells were considered colonies and were counted after 1 week. Experiments were performed in duplicate, and CFU-Fs were counted by 2 different operators. The efficiency of the ability to form colonies was calculated using a formula described previously [32].

The proliferation factor of hASCs was evaluated using a resazurin assay kit (TOX8, Sigma-Aldrich), following the manufacturers protocol. Test was performed on the 2nd, 5th, and 7th days of the experiment. Based on absorbance, the proliferation factor and PDT were calculated according to a previously described method [33].

### 2.6. hASCs Morphology

Growth pattern and cell morphology analysis was performed on the 7th day using an inverted, fluorescence microscope (AxioObserverA1, Zeiss) and a scanning electron microscope (SEM; EVO LS15, Zeiss). Evaluation of hASCs morphology included analysis of mitochondria, nuclei, and Golgi apparatus localization, as well as determination of cytoskeleton development. The mitochondria were stained using a rhodamine-based dye, MitoRed (Sigma-Aldrich, Germany), whereas the nuclei were stained using diamidino-2-phenylindole (DAPI). The cytoskeleton was dyed using atto-488-labeled phalloidin. Nuclei and cytoskeleton staining was performed after cells were fixed with 4% paraformaldehyde. Procedures involving fluorescence staining were performed in accordance with manufacturers' instructions and methods described previously.

After chondrogenic differentiation, cells were stained with 0.1% aqueous solution of Safranin O (specific for proteoglycans). Additionally, after osteogenic and adipogenic differentiation, the presence of calcium deposits and intracellular lipid vesicles was confirmed with Alizarin Red and Oil Red O staining, respectively.

Photographs were acquired using a PowerShot Camera (Cannon).

For SEM imaging, cells were prepared as was described earlier [34]. Briefly, postfixed cells were washed with distilled water and dehydrated in a graded series of ethanol dilutions (from 50% to 100%, every 10%). Dried samples were sputtered with gold and placed in the microscope chamber. Morphology of cells was determined using an SE1 detector at 10 kV of filament tension (SEM, Zeiss Evo LS 15).

### 2.7. Quantitative Collagen Types I and II, Aggrecan, Bone Morphogenetic Protein-2, Leptin, and Adiponectin Assays

The total concentration of chondrogenesis specific markers, Collagen type I (Col-I), Collagen type II (Col-II), Aggrecan (ACAN), and Bone Morphogenetic Protein-2 (BMP-2), was measured by enzyme-linked immunosorbent assay (ELISA) in collected supernatants derived from hASCs cultured in chondrogenic differentiation medium. In order to evaluate adipogenic differentiation efficiency, the concentration of adiponectin (ADIQ) and leptin (LEP) in the adipogenic differentiation medium was measured after 14 days of culture. Bone Morphogenetic Protein-2, ACAN, and Col-I ELISA kits were purchased from R&D Systems (R&D Systems, Abingdon, UK), and Col-II, ADIQ, and LEP kits were purchased from EIAab (Wuhan EIAab Science Co., China). The concentration of proteins was presented as a ratio of protein weight and supernatant volume (w/v).

### 2.8. Quantitative Reverse-Transcription Polymerase Chain Reaction

Expression of cartilage-specific markers (*Col-II* and* ACAN*), osteoblast-specific markers (*OPN*,* Col-I*,* OCL*, and* BMP-2*), and adipocyte specific-markers (*LEP*,* ADIQ*, and* PPAR-γ*) were analyzed by quantitative real-time PCR. Total RNA was isolated from cells by the phenol-chloroform method [35]. DNA-free RNA was prepared using DNase I RNase-free kit (Thermo Scientific, USA).

The primer sequences used in the study are shown in Table 1. CFX Connect*™* Real-Time PCR Detection System (Bio-Rad, USA) was used for PCR cycling. The PCR mixture contained 500 ng cDNA, 500 nM PCR primers, and Real-Time 2x PCR Master Mix SYBR B (A&A Biotechnology, Poland). The PCR reaction profile consisted of initial enzyme activation at 95°C for 2 min, followed by 45 cycles of denaturation at 95°C for 30 sec, annealing 30 sec with temperature dependent on the primer sequences (60°C* GAPDH*; 61.5°C* Col-I*; 67.1°C* BMP-2*; 64.8°C* OPN*; 67.5°C* OCL*; 60°C* LEP*; 60°C* ACAN*; 60°C* Col-II*; 60°C* ADIQ*; 60°C* PPAR-γ*), and extended at 72°C for 30 sec with a single fluorescence measurement. The series of cycles were followed by a melt curve analysis to ensure reaction specificity. The expression level of each gene was normalized to the housekeeping gene,* GAPDH*. Subsequently, the relative gene expression (Qn) was calculated in relation to the* GAPDH* gene [36].

### 2.9. Oxidative Stress and Senescence

Prior to assessing the level of oxidative stress, cells were cultured in normal DMEM growth medium without phenol red. Superoxide dismutase (SOD) activity was measured with the commercially available SOD Assay kit (Sigma-Aldrich, Germany), whereas the level of nitric oxide (NO) was estimated using Griess reagent kit (Life Technologies, USA). The production of ROS was determined by measuring H2DCF-DA (Life Technologies, USA). All procedures were performed in duplicate, according to the manufacturer's instructions.

As an indicator of senescence in cells,** SA-** *β*-galactosidase staining was performed, using Senescence Cells Histochemical Staining Kit (Sigma-Aldrich, Germany). After staining, according to the manufacturer's protocol, supernatants were collected and measured for absorbance at 420 nm. The number of viable cells and dead cells was assessed using Cell stain Double Staining Kit (Sigma-Aldrich, Germany). The nuclei of dead cells were stained with propidium iodide, whereas the nuclei of viable cells were stained with Calcein-AM. Cells were then observed using epifluorescence microscopy. The number of dead cells was calculated as percentage of all viable cells. All procedures were performed in accordance to manufacturer's instructions.

### 2.10. Statistical Analysis

Group data is presented as mean ± standard deviation (SD). Data analysis was performed using GraphPad Prism 5.0 (San Diego, USA). Statistical significance was determined using one-way analysis of variance (ANOVA) with Tukey's post hoc multiple comparison test. A *p* value of <0.05 was considered statistically significant.

## 3. Results

### 3.1. Donor Age Related Changes in hASCs Morphology and Proliferative Potential

**Figure 1:**
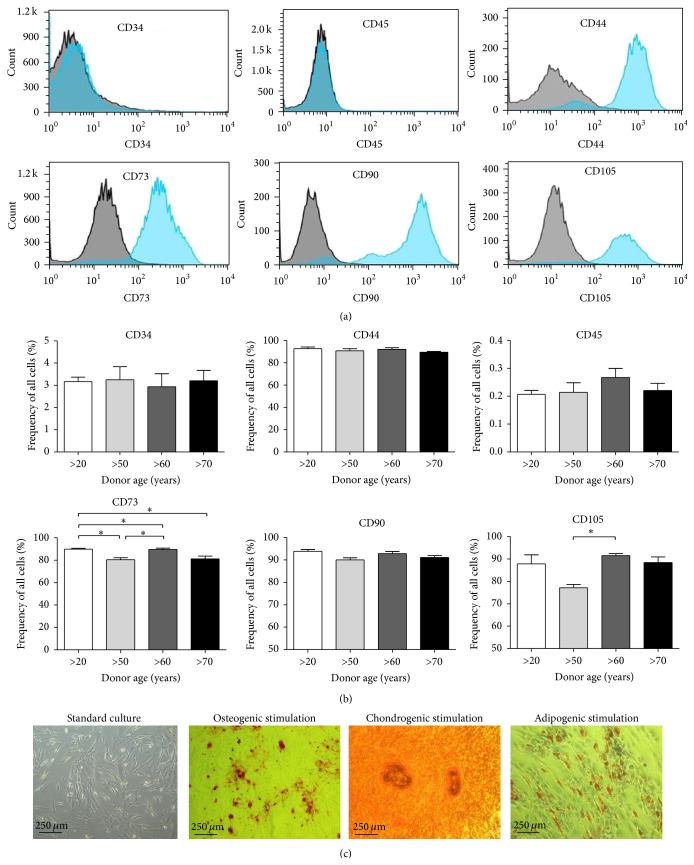
Characterization of MSCs. (a) Flow cytometry analysis of MSC positive (CD44, CD73b, CD90, and CD105) and negative (CD34 and CD45) surface markers. (b) Flow cytometry analysis determined the percentage of specific markers in total analyzed hASCs from patients differing in age. Quantification of markers revealed no significant differences between groups. (c) Alizarin Red staining of calcium deposits, Safranin O staining for proteoglycans, and accumulation of lipid droplets dyed with Oil Red O following differentiation into osteoblast, chondroblasts, and adipocyte lineages.

Human ASCs isolated from all four age groups fulfilled the MSC criteria: (i) typical plastic adherent growth; (ii) expression of CD44, CD73, CD90, and CD105 and absence of surface antigens CD34 and CD45 (Figures 1(a) and 1(b)); (iii) in vitro differentiation potential toward osteogenic, adipogenic, and chondrogenic lineages (Figure 1(c)). We did not observe any differences in regard to the percentage of MSC surface antigen expression, although there was some variation connected with donor age and CD73 expression (Figure 1(b)).

**Figure 2:**
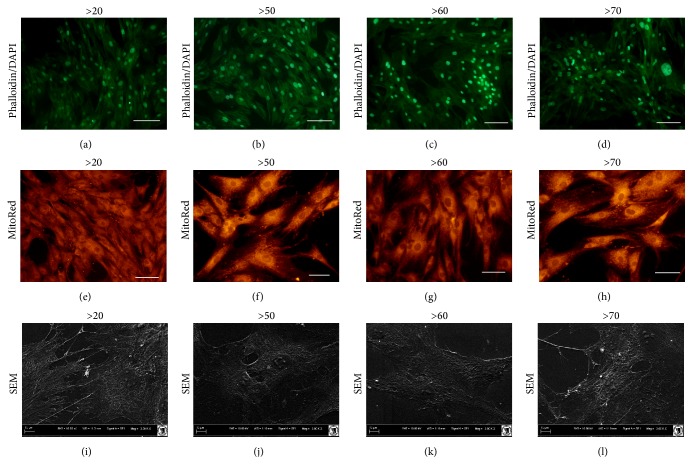
Comparison of hASCs morphology from different age groups, evaluated after seven days of propagation. Scale bars: (a–d) 250 *μ*m; (e–h) 300 *μ*m; (i–l) 10 *μ*m.

Plastic adherent colonies were observed in all donor samples. All cells displayed proper fibroblasts-like morphology, with predisposition to growth in close contact. Fluorescent staining after 7 days of culture indicated that all cells had the ability to grow in multilayer form, with centrally positioned nuclei. However, cells from donors >70 years were flatter than cells from other groups, with visible giant cells and signs of apoptosis (Figures 2(a)–2(d)). Additionally, the mitochondrial activity of MSCs from young and elderly patients was evaluated by MitoRed staining (Figures 2(e)–2(h)). We observed that mitochondria in young patients were evenly distributed within the cells, whereas with increased age they tended to be located mainly around the nuclei. Scanning electron microscopy analysis showed that hASCs obtained from donors in the >20 years age group were characterized by formation of a greater number of thin cellular projections (filopodia and lamellopodia) and several MVs located at the cell boundary (Figures 2(i)–2(l)).

**Figure 3:**
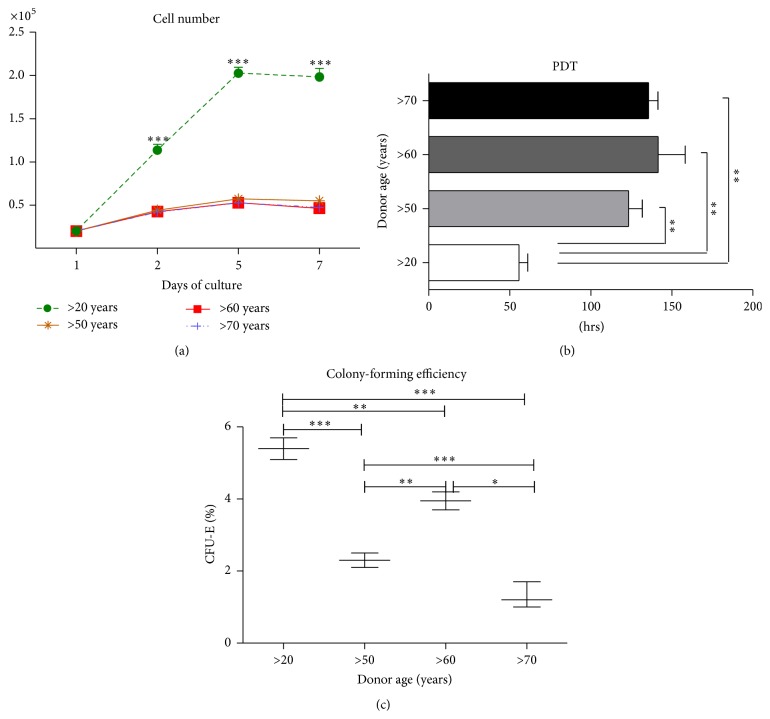
Comparison of cell proliferation in different age groups. (a) Cell proliferation assay, (b) population doubling time, and (c) fibroblast colony-forming unit efficiency over seven days. Results expressed as mean ± SD. ^*∗*^ *p* value <0.05.

Observations of cell growth kinetics revealed that the proliferation rate of cells obtained from young donors (>20 years of age) was significantly higher than in older patients, and the rate increased gradually until cells finally reached full confluence (day 5) (Figure 3(a)). Growth curves did not differ significantly between the older donor samples (>50, >60, and >70). Furthermore, there was a clear association between PDT and donor age (Figure 3(b)); cells from younger donors reached PDT faster than older patients. Between the older donor groups, we did not observe significant differences. Beyond the observation of a correlation between age and population doubling time, our results showed that the clonogenic potential of young and old MSCs samples was age-dependent. The percentage of CFU formation ranged from 0.9% (>70) to 5.7% (>20) (Figure 3(c)).

**Figure 4:**
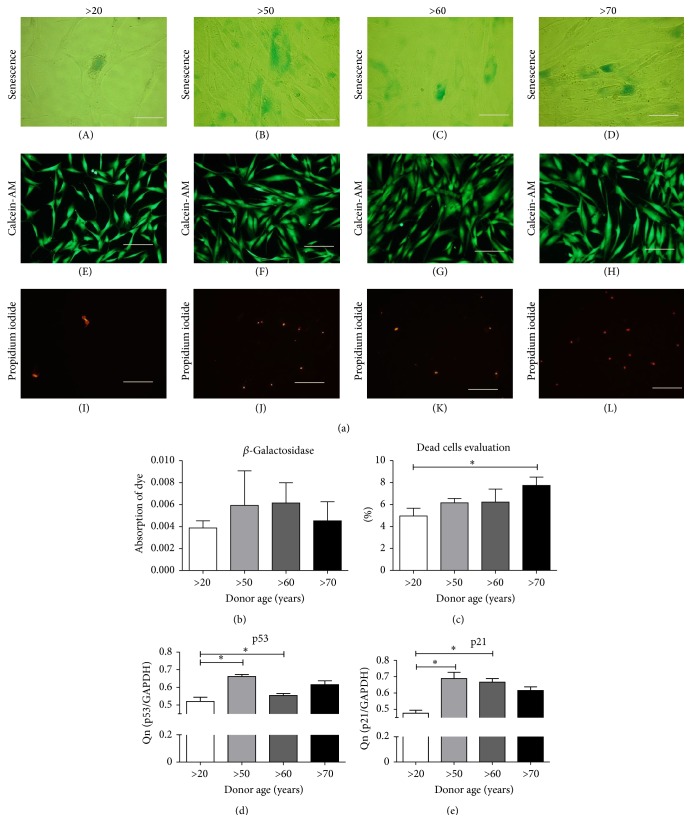
Characterization of age-dependent apoptosis. (a) Cells stained for senescence showing cells with *β*-galactosidase accumulation (A–D), Calcein-AM (E–H), and propidium iodide (I–L) for live-dead cells identification. (b) *β*-galactosidase absorbance measurement. (c) Percentage of apoptotic cells calculated from Calcein-AM/propidium iodide staining, (d) evaluation of p53, and (e) p21 on the mRNA level. Results expressed as mean ± SD. ^*∗*^ *p* value <0.05.

To validate that the decrease in cell growth results from age-dependent apoptosis, we calculated the number of dead cells (Figure 4(c)) marked with propidium iodide (Figure 4(a) (I)–(L)) and compared it to the number of live cells (Figure 4(a) (E)–(H)). We confirmed that with increasing donor age, the percentage of dead cells increases, although not to a level of statistical significance. We also confirmed the existence of a correlation between p53 and p21 mRNA levels in MSCs and the donor age (Figures 4(d) and 4(e)).

Moreover, after 7 days of culture, we observed that cells underwent replicative senescence. This was manifested by the expression of senescence associated beta-galactosidase, which was measured by dye absorption (Figure 4(b)). While the senescence of cells was more visible in older age groups through microscopic observations, absorption measurements indicated that even though younger cells showed lower dye absorption, the differences between groups were statistically insignificant.

**Figure 5:**
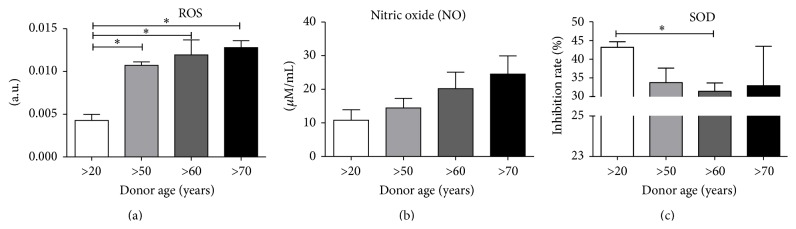
Levels of oxidative stress in hASCs. (a) Reactive oxygen species, (b) nitric oxide, and (c) superoxide dismutase levels. Results expressed as mean ± SD. ^*∗*^ *p* value <0.05.

Furthermore, we compared the oxidative stress senescence of cells and apoptosis. The obtained results revealed that the ROS level (Figure 5(a)) was significantly lower in young patients (>20) compared to older age groups. Interestingly, we did not detect significant differences in ROS levels between older age groups. The same relationship, also insignificant, was observed when comparing NO levels (Figure 5(b)). The lowest NO level was found in the >20 age group and the level increased with donor age. On the contrary, we observed only slight differences between the groups in SOD levels (Figure 5(c)).

### 3.2. The Ability of hASCs to Differentiate into Chondroblasts Decreases with Age

The ability of hASCs to differentiate into the chondrogenic cell lineage (samples from all four age groups) was evaluated based on lineage specific metabolite production, which was measured by protein and mRNA levels, as well as through observation using an inverted, fluorescence microscope and SEM.

**Figure 6:**
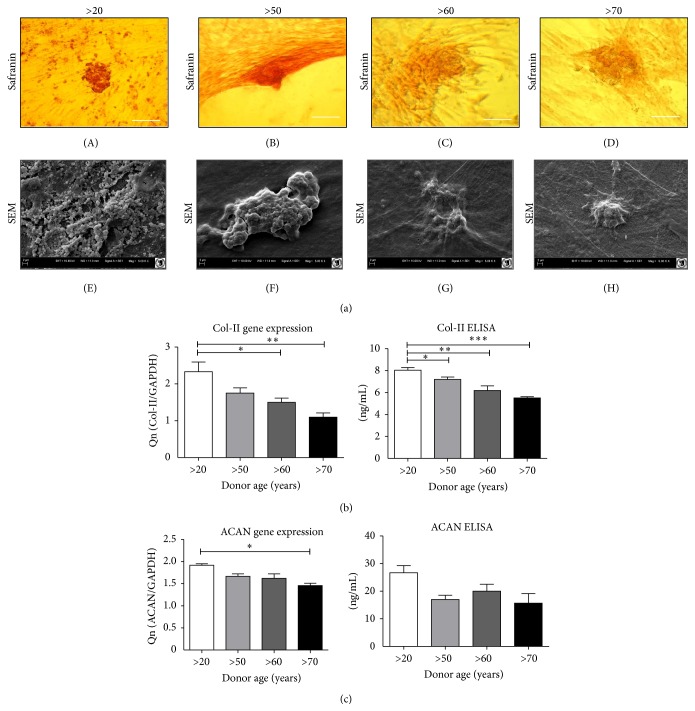
Characterization of age related changes in hASCs chondrogenic differentiation potential. (a) Morphology of cells cultured in chondrogenic medium visualized by Safranin O staining (A–D) and SEM (E–H). Quantitative ELISA analysis and gene expression of (b) Col-II and (c) ACAN. Results expressed as mean ± SD. ^*∗*^ *p* value <0.05.

At the beginning of the experiment, cells cultured in the chondrogenic differentiation medium displayed spindle-shape morphology, without any obvious age-dependent changes in cell shape or size. After 16 days of culture, chondro-e-like ASCs spontaneously formed large aggregates. Characteristic chondro-nodules were observed in all investigated cultures. Cells from the donors >20 years of age formed smaller aggregates, between which a cellular monolayer was created with the presence of fibroblast-like cells (Figure 6(a) (E)).

Histological staining of chondrogenic samples with Safranin O, after 16 days of induction, showed that the samples from all investigated donor groups formed nodules. The analysis of chondro-nodules in SEM showed that cells from older donors maintained a more spherical shape and had rare lamellopodia, whereas cells from the >20 years old group, formed loosely structured aggregates combined with several small chondro-nodules (Figure 6(a) (E)–(H)).

Results of qRT-PCR revealed significant age-related differences in the expression of genes encoding cartilage matrix: Col-II and ACAN (Figures 6(b) and 6(c)). We found significant differences between the >20 years old group and older patients, whereas we did not observe significant changes in ACAN expression between elderly patients. Expression levels of all matrix proteins were highest in young patients and decreased with donor age. Quantitative evaluation of Col-II and ACAN on an mRNA level was additionally confirmed on the protein level by an ELISA test (Figures 6(b) and 6(c)). In contrast to gene expression analysis, we did not observe significant changes between groups in ACAN secretion. However, analysis of the concentration of Col-II on the protein level indicated a significant decrease of Col-II concentration with increasing donor age.

## 4. Osteogenic Differentiation Potential of hASCs Decreases with Donor Age

**Figure 7:**
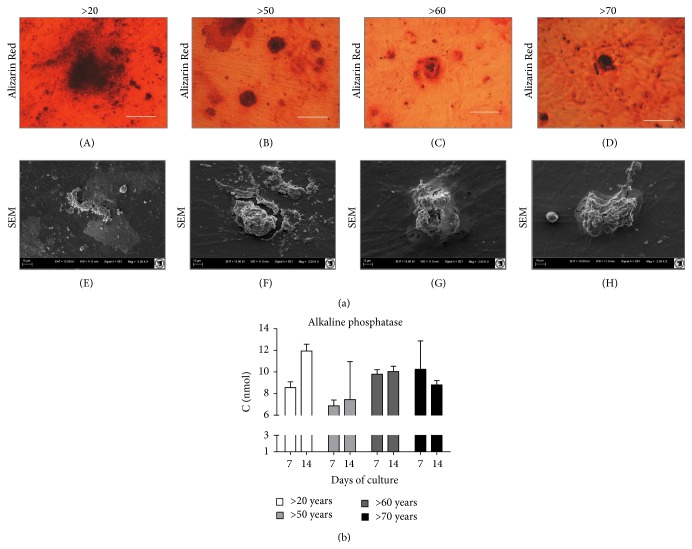
Characterization of age related changes in hASCs osteogenic differentiation potential. (a) Morphology of cells cultured in osteogenic medium visualized by (A–D) Alizarin Red staining and SEM (E–H). (b) SEM quantitative evalutaion of ALP secretion.

**Figure 8:**
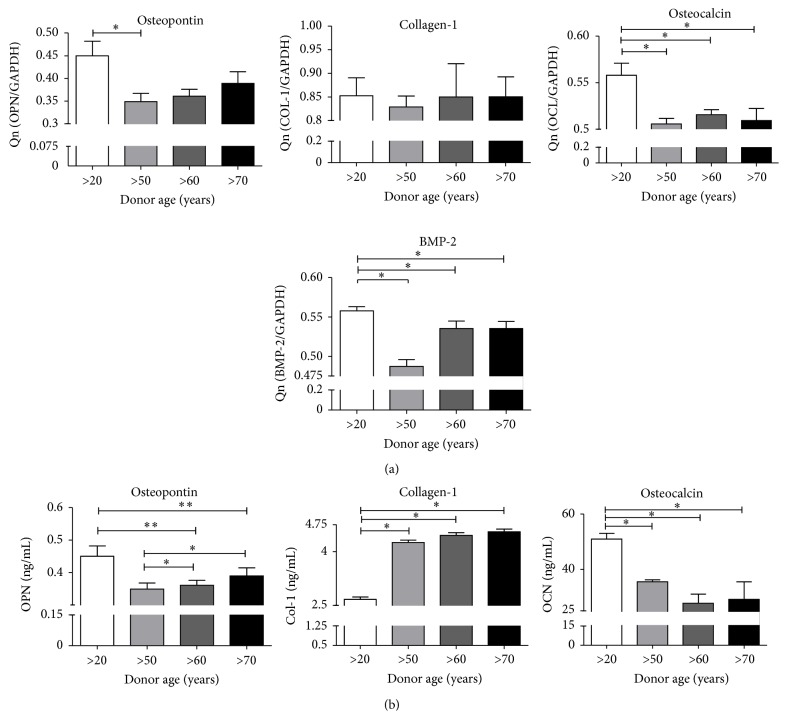
Gene expression and ELISA anaylsis of osteogenic markers. (a) Gene expression analysis and (b) quantitative ELISA analysis of* OPN*,* Col-I*, and* OCL*. Results expressed as mean ± SD. ^*∗*^ *p* value <0.05.

Osteogenic differentiation of young and old hASCs was evaluated based on extracellular matrix calcification. Mineral calcium deposits and hydroxyapatite-like structures were visualized by Alizarin Red staining and observed in all investigated groups. Noticeably greater nodules were observed in ASCs from younger donors. This coincided with the results of calcium and phosphorus content in bone nodules, measured by SEM-EDX (Figure 7(a)). Alkaline phosphatase activity in cells measured after 7 days and after 14 days was highly variable between groups, but did not display any significant changes (Figure 7(b)). No significant correlation between age and hASC differentiation capacity was observed. Gene expression analysis of OPN, OCL, and BMP-2 revealed higher expression of all investigated osteogenic markers in younger patients, with only slight differences between older groups (Figure 8(a)). An exception was the expression of Col-I, where we observed a similar trend for all donor groups. Similarly, on the protein level, we observed a higher concentration of secreted OPN, OCN, and BMP-2 in young patients and a higher concentration of Col-I in the elderly (Figure 8(b)).

### 4.1. Age-Related Changes in hASC Differentiation into Adipocytes

**Figure 9:**
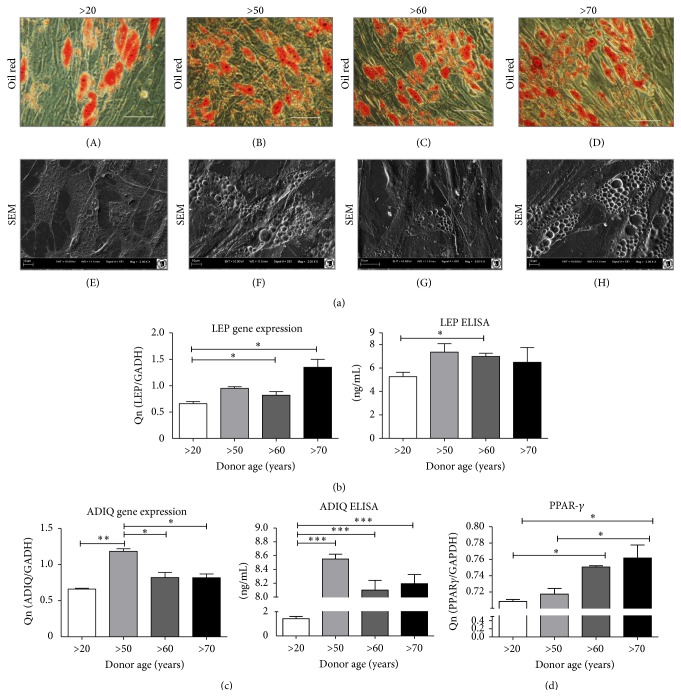
Characterization of age related changes in hASCs adipogenic differentiation potential. (a) Morphology of cells cultured in adipogenic medium visualized by Oil Red O staining (A–D) and SEM (E–H). Quantitative ELISA analysis and gene expression of* LEP* (b) and* ADIQ* (c). Gene expresion analysis of* PPAR-γ* (d). Results expressed as mean ± SD. ^*∗*^ *p* value <0.05.

Qualitative assessment of Oil Red O staining revealed that, after 14 days, adipogenic induced hASCs populations produced similar amounts of lipid droplets in all investigated groups (Figure 9(a)). We did not find differences between the groups in the number and size of lipid vacuoles dyed by Oil Red O (Figure 9(a) (A)–(D)). However, slight differences between groups were visible under SEM, through which the youngest donor group appeared to have a smaller amount of lipid droplets. Moreover, the differentiation response was reduced in the >20 years old group, which was manifested by lower adiponectin and leptin levels, measured by both ELISA (Figure 9(b)) and qRT-PCR (Figure 9(c)). Furthermore, we observed an age-dependent increase in the mRNA level of PPAR-*γ* gamma expression (Figure 9(d)).

## 5. Discussion

The deterioration of the regenerative potential upon aging has been suspected to be due to functional changes in adult stem cells. To confirm this hypothesis, we investigated several distinct parameters including growth curve, proliferation factor, PDT, and differentiation capacity into chondrogenic, osteogenic, and adipogenic lineage in hASCs derived from different donor age groups.

In this study, differences among hASC populations derived from >20, >50, >60, and >70 years old donor groups were manifested by cell expansion properties. Our results found that age significantly affected the growth kinetic and the PDT of the investigated groups, with evident differences between young and elderly patients. Interestingly, there were no significant changes in regard to proliferation activity between the older patient groups. This observation could suggest that while proliferative activity of cells decreases with age, hASCs do not completely lose their proliferative potential, even in the elderly. However, in clinical practice, due to the lower proliferative rates of cells isolated from older patients, it may be necessary to culture and expand MSCs in vitro for a longer period before clinical use, which may lengthen the time needed for clinical autologous application in these patients.

Our data is consistent with results of other studies that were conducted on bone marrow derived MSCs, which showed that aging slowed the PDT [37, 38]. In correlation with growth curve and PDT changes, we observed an age-dependent reduction in the ability to form single cell-derived colonies. The number of colony-forming MSCs strongly correlates with both their culture expansion ability and their differentiation potential. Taking into account the proliferation rate, the PDT, and the CFU of cells is important for their clinical application due to the large number of cells necessary for therapy.

A reduction in proliferation rate and viability of cells might be ultimately reflected in cell senescence and apoptosis [39]. MSCs can undergo only a limited number of cell divisions in a process called cellular senescence. Senescence is considered to be a stress response triggered by the activation of p53 and is highly influenced by oxidative stress [40]. In our study, we observed age-dependent senescence manifested by an altered state of growth, which correlates with decreasing mitochondrial activity, high levels of p53 and p21, and increased activity of *β*-galactosidase. Age-dependent intensification of senescence has been shown in animal models and confirms that with increasing donor age, the number of senescent cells accumulating *β*-galactosidase is also increasing [41, 42]. Moreover, based on gene expression analysis, there is experimental evidence suggesting that aging involves the p53 and p21 pathway. The p21 protein is a substrate for caspase-3 activity which appears to be activated by ROS, thereby facilitating apoptotic response [43, 44]. In our experiment, cells derived from older groups had elevated concentrations of ROS and NO and simultaneously decreased levels of SOD, which plays an important role as an antioxidative factor in the cell [45].

Multilineage differentiation potential has been considered an important quality of MSCs [46]. Although it has been shown that adult human MSCs can differentiate into the chondrogenic lineage, little is known about whether this differentiation capacity is age-dependent. While our results show that age reduces chondrogenic differentiation potential of hASCs, this reduction does not necessarily indicate that the cells have lost their regenerative potential. In all investigated groups we clearly observed the proteoglycan-rich extracellular matrix and the typical round chondro-like cell morphology, but there were significant differences in concentration of chondrogenesis markers on protein and mRNA levels. We observed mRNA upregulation in regards to cartilage-specific genes Col-II and ACAN. However, there were significant differences between age groups, favoring the youngest donor group. This is similar to findings of Choudhery et al. [24], who also observed that the capacity of hASCs chondrogenic potential declines with age. Age-related changes of MSCs chondrogenic potential were investigated previously in both human and animal studies; however, these studies were performed mostly on bone marrow stromal cell (BSMC) models and have reported conflicting results. Murphy et al. [47] reported an age-related decrease in chondrogenic differentiation of human BSMCs. In rodent and rabbit models, chondrogenesis of BMSCs confirmed an age-related decrease in the capacity to differentiate [38, 41]. However, De Bari et al. [48] did not observe any correlation between age and chondrogenic differentiation of multipotent cells isolated from adult human synovial membranes.

In this study, age-related osteogenic differentiation capacity was consistent with chondrogenic differentiation potential. We observed an age-related downregulation of cells osteogenic potential. We also detected that cells derived from young donors had an increased expression of osteogenic markers (BMP-2, OPN, and OCL) and were able to form osteonodules with a higher content of calcium deposits. Our findings related to MSCs osteogenic potential are supported by other studies. Choudhery et al. [24] showed a significant decrease in osteogenic potential of hASCs, which was also manifested by a decrease in osteocalcin and ALP gene expression in elderly donors. These observations are in line with the work of Zhu et al. [17], who reported a decrease in the osteogenic potential with increasing age. However, no aging effects were observed when comparing early and late osteogenesis markers, as well as calcified matrix deposition for young and old BMSCs [17]. These discrepancies could be due to the fact that different cells were used in the previously mentioned experiments, or the different age ranges and different health statuses of the cell donors.

Due to the fact that degenerative bone and joint diseases increase in prevalence with age, the age-related decrease in both chondrogenic and osteogenic differentiation potential may be a limitation for the therapeutic applications of hASCs. Future studies should assess ways to overcome the limitations in age-related differentiation capacity. Furthermore, Matsumoto et al. [49] reported sex based differences in chondrogenic differentiation and cartilage regeneration potential in MSCs derived from muscle tissue. Muscle-derived stem cells (MDSCs) from men displayed greater chondrogenic differentiation and better cartilage repair as compared to MDSCs from females [49]. Combined with the effects of aging, sex based differences present a further potential limitation to clinical application of MSCs. As such, future studies should also assess sex based differences in chondrogenic differentiation capacities of hASCs.

In regard to age related adipogenic differentiation, there were conflicting observations. On the one hand, we did not observe differences between age groups in the quantity of lipid droplets accumulation, but on the other hand we have found that leptin, adiponectin, and PPAR-*γ* concentrations were elevated in older patients as compared to the >20 years old group. It is well known that MSCs may lose their adipogenic activity with donor aging and that this may be associated with the development of disease and abnormal stem cell activity. In vitro research conducted on BMSCs demonstrated loss of MSC osteogenic and adipogenic potential with aging [50, 51]. We can clearly state that MSC aging shifts the differentiation balance in favor of adipogenic differentiation and decrease osteogenic and chondrogenic differentiation potential. This was confirmed by the observations of PPAR-*γ* expression in our experiment. Activation of PPAR-*γ* is associated with increased adipocyte differentiation that subsequently promotes weight gain and obesity. PPAR-gamma suppresses the differentiation of osteoblasts progenitors, while shifting cells in favor of adipogenic differentiation [52]. This subsequently may result in decreased osteoclastogenesis and bone remodeling [52]. In general, the available literature suggests an overall decrease in differentiation potential of MSCs with donor age, regardless of species.

Understanding the influence of age on MSCs properties is important due to their potential therapeutic use in musculoskeletal disorders that are widespread among the elderly. Our results demonstrate that not only the proliferation status of MSCs, but also their multilineage differentiation capacity, may provide an argument to restrict MSC-based therapies to certain individuals. Future studies comparing the effects of aging on different MSCs populations could help to optimize treatments by identifying an MSC source that is less adversely affected by age.

## Figures and Tables

**Table 1:** Primer sequences used in qPCR.

Gene	Primer 5′-3′	Amplicon length (bp)	Accession number
ACAN	F: GCCTACGAAGCAGGCTATGA	136	NM_013227.3
	R: GCACGCCATAGGTCCTGA		

ADIQ	F: AGGGTGAGAAAGGAGATCC	4155	XM_011513324.1
	R: GGCATGTTGGGGATAGTAA		

BMP-2	F: ATGGATTCGTGGTGGAAGTG	349	KC294426.1
	R: GTGGAGTTCAGATGATCAGC		

COL-I	F: GTGATGCTGGTCCTGTTGGT	123	NM_000088.3
	R: CACCATCGTGAGCCTTCTCT		

COL-II	F: GACAATCTGGCTCCCAAC	257	NM_001844.4
	R: ACAGTCTTGCCCCACTTAC		

GAPDH	F: GTCAGTGGTGGACCTGACCT	286	NM_0017008.4
	R: CACCACCCTGTTGCTGTAGC		

LEP	F: ATGACACCAAAACCCTCATCAA	22	XM_005250340.3
	R: GAAGTCCAAACCGGTGACTTT		

OCL	F: ATGAGAGCCCTCACACTCCTC	292	NM_199173.4
	R: CGTAGAAGCGCCGATAGGC		

OPN	F: AAACGCCGACCAAGGTACAG	213	U20758.1
	R: ATGCCTAGGAGGCAAAAGCAA		

PPAR*γ*	F: ATGACACCAAAACCCTCATCAA	125	AB565476.1
	R: GAGCGGGTGAAGACTCATGTCTGTC		
